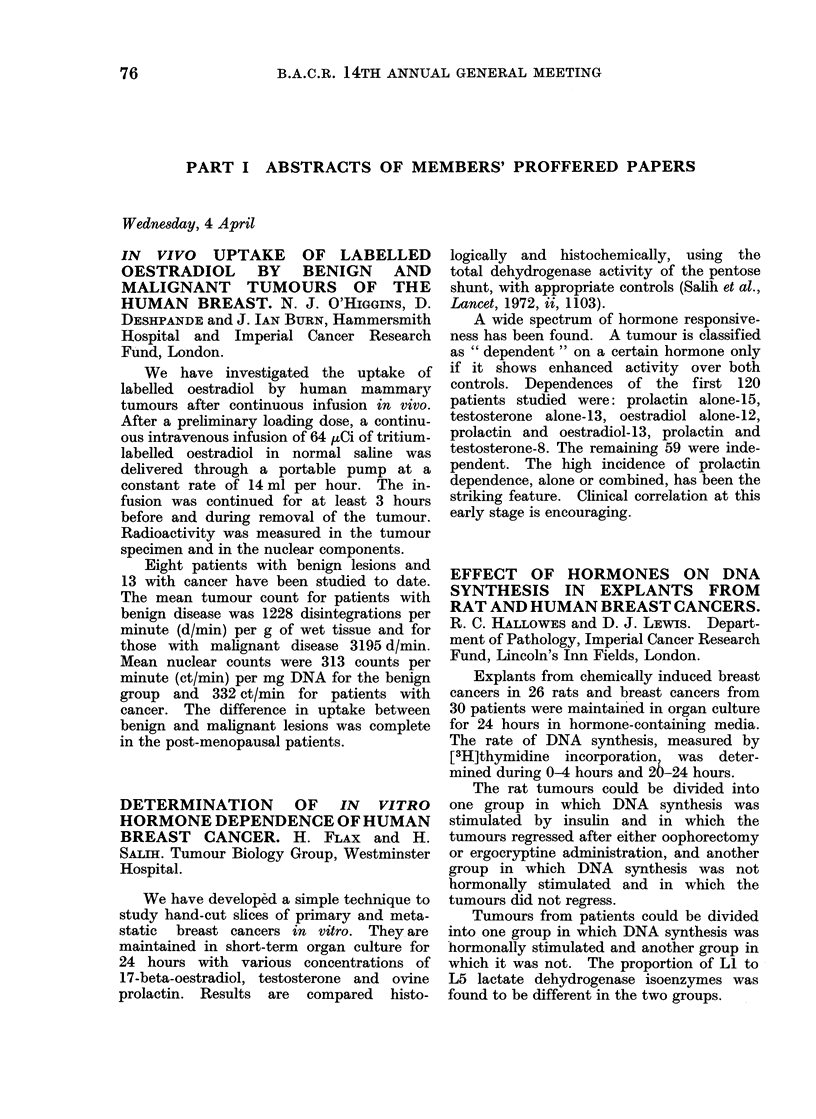# Determination of in vitro hormone dependence of human breast cancer.

**DOI:** 10.1038/bjc.1973.74

**Published:** 1973-07

**Authors:** H. Flax, H. Salih


### DETERMINATION OF *IN VITRO* HORMONE DEPENDENCE OF HUMAN BREAST CANCER.

H. Flax and H. Salih. Tumour Biology Group, Westminster Hospital.

We have developed a simple technique to study hand-cut slices of primary and metastatic breast cancers *in vitro*. They are maintained in short-term organ culture for 24 hours with various concentrations of 17-beta-oestradiol, testosterone and ovine prolactin. Results are compared histologically and histochemically, using the total dehydrogenase activity of the pentose shunt, with appropriate controls (Salih *et al.*, *Lancet*, 1972, *ii*, 1103).

A wide spectrum of hormone responsiveness has been found. A tumour is classified as " dependent " on a certain hormone only if it shows enhanced activity over both controls. Dependences of the first 120 patients studied were: prolactin alone-15, testosterone alone-13, oestradiol alone-12, prolactin and oestradiol-13, prolactin and testosterone-8. The remaining 59 were independent. The high incidence of prolactin dependence, alone or combined, has been the striking feature. Clinical correlation at this early stage is encouraging.